# Machine learning based predictive model and genetic mutation landscape for high-grade colorectal neuroendocrine carcinoma: a SEER database analysis with external validation

**DOI:** 10.3389/fonc.2025.1509170

**Published:** 2025-01-29

**Authors:** Ruixin Wu, Sihao Chen, Yi He, Ya Li, Song Mu, Aishun Jin

**Affiliations:** ^1^ Department of Immunology, School of Basic Medical Sciences, Chongqing Medical University, Chongqing, China; ^2^ Chongqing Key Laboratory of Tumor Immune Regulation and Immune Intervention, Chongqing, China; ^3^ Department of Gastrointestinal Surgery, the First Affiliated Hospital of Chongqing Medical University, Chongqing, China; ^4^ Department of Colorectal Surgery, The Affiliated Hospital of Guizhou Medical University, Guiyang, Guizhou, China

**Keywords:** high-grade colorectal neuroendocrine carcinoma (HCNEC), machine learning, prognosis, SEER, COSMIC, genetic mutation landscape

## Abstract

**Background:**

High-grade colorectal neuroendocrine carcinoma (HCNEC) is a rare but aggressive subset of neuroendocrine tumors. This study was designed to construct a risk model based on comprehensive clinical and mutational genomics data to facilitate clinical decision making.

**Methods:**

A retrospective analysis was conducted using data from the Surveillance, Epidemiology, and End Results (SEER) database, spanning 2000 to 2019. The external validation cohort was sourced from two tertiary hospitals in Southwest China. Independent factors influencing both overall survival (OS) and cancer-specific survival (CSS) were identified using LASSO, Random Forest, and XGBoost regression techniques. Molecular data with the most common mutations in CNEC were extracted from the Catalogue of Somatic Mutations in Cancer (COSMIC) database.

**Results:**

In this prognostic analysis, the data from 714 participants with HCNEC were evaluated. The median OS for the cohort was 10 months, whereas CSS was 11 months. Six variables (M stage, LODDS, Nodes positive, Surgery, Radiotherapy, and Chemotherapy) were screened as key prognostic indicators. The machine learning model showed reliable performance across multiple evaluation dimensions. The most common mutations of CNEC identified in the COSMIC database were TP53, KRAS, and APC.

**Conclusions:**

In this study, a refined machine learning predictive model was developed to assess the prognosis of HCNEC accurately and we briefly analyzed its genomic features, which might offer a valuable tool to address existing clinical challenges.

## Introduction

Neuroendocrine tumors, which are rare malignancies, arise from peptidergic neurons and neuroendocrine cells (1, 2). Advances in diagnostic techniques such as endoscopy and hematological markers have contributed to a marked increase in the detection of neuroendocrine cancer (3, 4). These tumors can manifest across various body sites, including the digestive system, particularly the colorectum, which is a prevalent location, and they exhibit notable heterogeneity (5, 6). According to the World Health Organization (WHO) classification criteria of 2019, neuroendocrine tumors are classified as well-differentiated neuroendocrine tumors (NETs), poorly differentiated neuroendocrine carcinomas (large cell/small cell, NECs), and mixed neuroendocrine-non-neuroendocrine tumors (MiNENs) (7). While existing research predominantly addresses well-differentiated NETs, there remains a research gap concerning more aggressive high-grade colorectal neuroendocrine carcinoma (HCNEC). HCNEC’s elusive onset and intricate pathological classification often lead to clinical misdiagnoses, resulting in an advanced-stage diagnosis for the majority of patients (8, 9). As a result, the median survival duration for these patients is typically less than 1 year (10, 11).

For non-metastatic patients, a combination of surgery and postoperative systemic therapy is the most potent therapeutic approach, although the rate of successful radical surgical resection remains suboptimal (12). For patients with metastatic or unresectable conditions, the prevalent clinical interventions include vascular interventional embolization, local ablation, systemic chemotherapy, peptide receptor radionuclide therapy, and targeted radiotherapy (13, 14). However, the outcomes of these interventions have been constrained. Established management guidelines and precise tumor staging play pivotal roles in clinical decision-making. Regrettably, current references for HCNEC predominantly align with those for colorectal adenocarcinoma, revealing the absence of standardized clinical guidelines and a dedicated prognostic evaluation system.

Given the pressing clinical requirements, our study introduced and validated an interactive machine learning survival prediction model based on extensive population data. Furthermore, we have analyzed the mutated genes in this rare tumor, which has helped us understand its genetic landscape. This aim extends beyond filling the void in existing prognostic frameworks to further solidify the groundwork for clinical decision-making.

## Methods

### Study design and selection criteria

**Figure 1:**
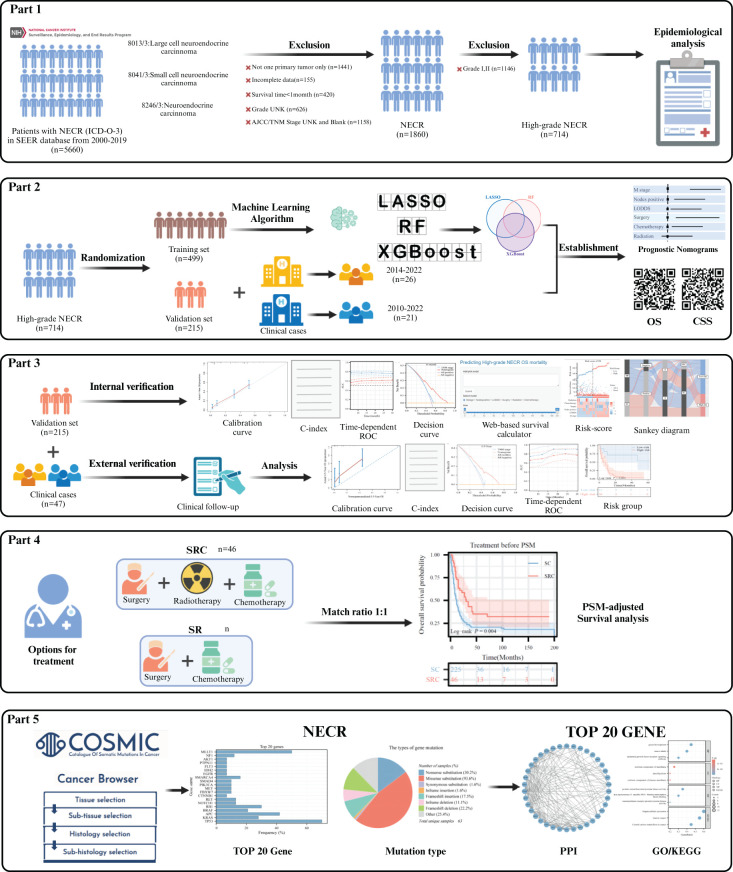
Study design and the workflow diagram.

This study adhered to the Transparent Reporting of a Multivariable Prediction Model for Individual Prognosis or Diagnosis (TRIPOD) reporting guidelines for prognostic studies. The comprehensive workflow is shown in **Figure 1**. Ethical considerations adhered to the 2013 revised Declaration of Helsinki and received approval from the Ethics Committee of the Affiliated Hospital of Guizhou Medical University, China (Approval No. 2023-630), as well as the First Affiliated Hospital of Chongqing Medical University, China (Approval No. 2024-086). Informed consent was obtained from all subjects. Public data were sourced from the SEER database, a significant open-access repository.

Incidence data were acquired using the SEER*Stat software [Incidence - SEER 17 Regs Research Data, Nov 2022 Sub (2000-2020)]. Incidence rates were adjusted relative to the age of the standard American population, as of 2000. Temporal trends across the three pathological types and the age-sex distribution characteristics were also analyzed. Complete follow-up and treatment data were collected from the Incidence-SEER 17 Regs Research Plus Data, Nov 2021 Sub (2000-2019) and the screening phase utilized the following specific criteria: only patients diagnosed with HCNEC (primary site: C18.0-C18.9, C19.9, C20.9) possessing codes 8013/3: large cell neuroendocrine carcinoma, 8041/3: small cell carcinoma, and 8246/3: neuroendocrine carcinoma, NOS, with grade 3/4 (poorly differentiated/undifferentiated) from 2000 to 2019 were included. These records indicated a unique primary tumor. The recorded survival duration for patients must be a minimum of one month. Each patient’s dataset required a comprehensive follow-up. The essential data elements for each patient included vital status, survival duration, demographics (age, sex, and race), number of positive lymph nodes (PLNs), count of dissected lymph nodes (DLNs), pathological grade, 7th T/N/M stage, CS tumor dimensions, and primary therapeutic approaches. The primary observation endpoint of the study was OS, defined as the time from diagnosis until death for any reason and the cancer-specific survival (CSS) was defined as the time from diagnosis of the study until death for tumor only). The Log odds of positive lymph nodes (LODDS) were determined with the following expression:


Log[(PLNs+0.5)/(DLNs−PLNs+0.5)]

To differentiate cancer-specific from non-cancer-specific survival outcomes in HCNEC, SEER variables pertinent to cause-specific death classifications and other causes of mortality were employed. Relevant treatment data, spanning the sequence of radiation post-surgery to the rationale for oncology-focused surgical procedures, radiation recording, and chemotherapy recording, were extracted from the respective fields. For external validation, we enrolled 26 patients with HCNEC treated at the First Affiliated Hospital of Guizhou Medical University from 2014 to 2022 and 21 patients with HCNEC treated at the First Affiliated Hospital of Chongqing Medical University from 2010 to 2022. Six samples (3 tumor tissues and paired 3 adjacent non-cancerous tissues) from 3 HCNEC patients from the First Affiliated Hospital of Chongqing Medical University were used to extract total RNA. cDNA was synthesized by reverse transcription, and RT-qPCR was performed using a qPCR kit (Takara Bio) to determine gene expression. The PCR primer sequences are shown in **Supplementary Table 1**. GAPDH was used as a control standard and was calculated using the relative mRNA-Δ;Δ;Ct method for comparison.

### Statistical analysis

Data from the SEER database were randomly partitioned into training and validation subsets in a 7:3 ratio. Age-adjusted incidence rates were computed as per 100,000 individuals using the SEER statistic, and annual percentage changes (APCs) were also determined. Categorical variables were evaluated by computing frequencies and are presented as percentages. Their significance was ascertained using the chi-square test. Survival trends were delineated using the Kaplan-Meier method, with disparities among the curves identified using the log-rank test. The restricted cubic spline (RCS) method was employed to establish cutoff values for the LODDS and DLNs (**Supplementary Figure 1**). Factors influencing OS and CSS were identified using regression analyses, specifically the Least Absolute Shrinkage and Selection Operator (LASSO), Random Forest (RF), and extreme Gradient Boosting (XGBoost) algorithms. Common features across the three algorithms were chosen as the definitive variables for the nomograms, which subsequently served as foundational elements in the digital survival risk-prediction model. To enhance the transparency and interpretability of the model, the SHAP method was employed to interpret the predicted results.

For model discrimination, the area under the time-dependent ROC was assessed, complemented by the C-index. Calibration plots were constructed to juxtapose the predicted survival rates with observed outcomes. In contrast to TNM stage, the predictive accuracy of the model was ascertained using both DCA and time-dependent ROC. Individualized risk scores were derived by employing the constructed nomograms and categorizing patients into higher- or lower-risk groups. The Surv_Cutpoint function was used to pinpoint the optimal cutoffs for OS and CSS. Heatmaps visually displayed risk factor associations and illustrated the distribution of clinical features among various risk categories for OS and CSS. Sankey diagrams were generated for each variable in the final risk category to enhance the clinical relevance of the framework. To ensure a meticulous comparison of survival rates across various treatments, we integrated Propensity Score Matching (PSM) analysis (1:1 ratio). The top 20 mutated genes derived from the COSMIC database were utilized for subsequent PPI network analysis (Confidence score > 0.7) and imported into Cytoscape software (v3.8.2) for visualization. For biological process and pathway enrichment analyses, the Kyoto Encyclopedia of Genes and Genomes (KEGG) and Gene Ontology (GO) analyses were performed using the R clusterProfiler package. Our analytical methods hinged on SPSS (version 26.0), R software (version 4.1.1), and Python (version 3.7), all findings were deemed significant at two-sided P values less than 0.05. All images were produced using Adobe Illustrator 2024 software.

## Results

### Epidemiological characteristics analysis

**Figure 2:**
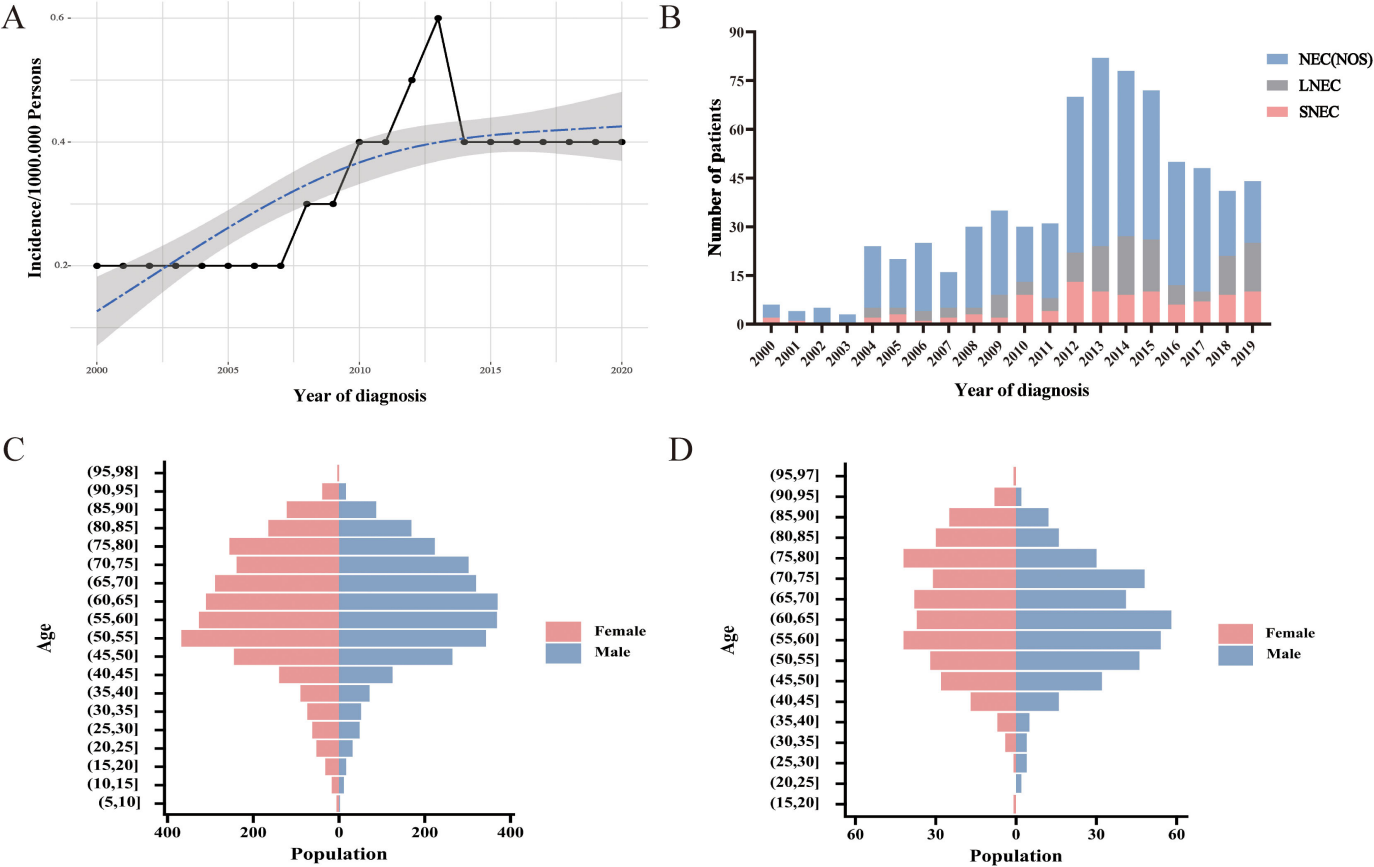
Trends and characteristics of CNEC. **(A)** Incidence of CNEC from 2000 to 2020. **(B)** Temporal changes in the proportions of three pathological types. **(C)** Age-sex distribution in CNEC. **(D)** Age-sex distribution in HCNEC.

The incidence of CNEC consistently increased between 2000 and 2020, with an APC of 4.1% (95% CI:1.9-6.3; *P*< 0.05) (**Figure 2A**). Among the three subtypes, neuroendocrine carcinoma (NOS) was the predominant pathological type (**Figure 2B**). Both CNEC and HCNEC displayed approximately uniform distributions in terms of age and sex (**Figures 2C, D**).

### Clinical characteristics of patients

Data from 714 individuals diagnosed with HCNEC between 2000 and 2019 were sourced from the SEER database. The cohort was stratified into a 7:3 split, designating 499 individuals as the training set and 215 as the validation set. Clinical characteristics were evaluated to identify any disparities between subsets, revealing no significant differences (P > 0.05) in demographic or clinical attributes. Demographic and clinical data are summarized in **Table 1**. Key findings included that the majority of participants were aged >60 years (n=419, 58.7%), primarily Caucasian (n=604, 84.6%), with tumor size ≥2 cm (n=556, 77.9%), exhibiting poorly differentiated pathological features (n=492, 68.9%), neuroendocrine carcinoma (NOS) (n=490, 68.6%), and from lower-income households (n=499, 69.9%). Regarding treatment, 68.1% underwent gross total resection (GTR) or subtotal resection (STR), 63.2% received chemotherapy, and 18.2% underwent radiotherapy. The overall median survival was 10 months (range:8.7-11.3), and the median cancer-specific survival was 11 months (range:9.7-12.3). Specifically, the training set showed a median OS of 10 months (range:8.6-11.4) and CSS of 11 months (range:9.6-12.4), while the validation set reported 10 months (range:7.3-12.6) and 10 months (range:6.8-13.2), respectively. Moreover, 26 patients with HCNEC treated at the First Affiliated Hospital of Guizhou Medical University and 21 patients with HCNEC treated at the First Affiliated Hospital of Chongqing Medical University were included for external validation. This external cohort had a median OS of 8 months (range:3.3-12.7) and 10 months (range:5.5-14.6), with demographic and clinical details provided in **Supplementary Table 2**.

**Table 1:** Characteristics of patients with HCNEC in the training and validation cohort.

Characteristics	Total (n=714)	Training cohort (n=499)	Validation cohort (n=215)	*P value*
	no.(%)	no.(%)	no.(%)	
Year of diagnosis				0.760
2000-2009	168 (23.5%)	119 (23.8%)	49 (22.8%)	
2010-2019	546 (76.5%)	380 (76.2%)	166 (77.2%)	
Gender				0.673
Male	370 (51.8%)	243 (48.7%)	101 (47.0%)	
Female	344 (48.2%)	256 (51.3%)	114 (53.0%)	
Age				0.967
<50	106 (14.8%)	75 (15.0%)	31 (14.4%)	
50-60	189 (26.5%)	131 (26.3%)	58 (27.0%)	
>60	419 (58.7%)	293 (58.7%)	126 (58.6%)	
Marital status				0.388
Married	391 (54.8%)	268 (53.7%)	123 (57.2%)	
Others	323 (45.2%)	231 (46.3%)	92 (42.8%)	
Household income				0.266
≥75000$	215 (30.1%)	144 (28.9%)	71 (33.0%)	
<75000$	499 (69.9%)	355 (71.1%)	144 (67.0%)	
Race				0.155
White	604 (84.6%)	430 (86.2%)	174 (80.9%)	
Black	65 (9.1%)	39 (7.8%)	26 (12.1%)	
Others	45 (6.3%)	30 (6.0%)	15 (7.0%)	
Histological				0.897
SNEC	102 (14.3%)	73 (14.6%)	29 (13.5%)	
LNEC	122 (17.1%)	86 (17.2%)	36 (16.7%)	
NEC (NOS)	490 (68.6%)	340 (68.1%)	150 (69.8%)	
Grade				0.464
Poorly differentiated	492 (68.9%)	348 (69.7%)	144 (67.0%)	
Undifferentiated	222 (31.1%)	151 (30.3%)	71 (33.0%)	
Primary Site				0.279
LSC	87 (12.2%)	70 (14.0%)	21 (9.8%)	
RSC	398 (55.7%)	273 (54.7%)	121 (56.2%)	
Rectal	229 (32.1%)	156 (31.3%)	73 (34.0%)	
T stage				0.329
T1	164 (23.0%)	115 (23.0%)	49 (22.8%)	
T2	94 (13.2%)	71 (14.2%)	23 (10.7%)	
T3	256 (35.8%)	182 (36.5%)	74 (34.4%)	
T4	200 (28.0%)	131 (26.3%)	69 (32.1%)	
N stage				0.217
N0	248 (34.7%)	170 (34.1%)	78 (36.1%)	
N1	341 (47.8%)	248 (49.7%)	93 (43.3%)	
N2	125 (17.5%)	81 (16.2%)	44 (20.5%)	
M stage				0.318
M0	369 (51.7%)	264 (52.9%)	105 (48.8%)	
M1	345 (48.3%)	235 (47.1%)	110 (51.2%)	
Clinical stage				0.598
I	47 (6.6%)	31 (6.2%)	16 (7.4%)	
II	87 (12.2%)	64 (12.8%)	23 (10.7%)	
III	235 (32.9%)	169 (33.9%)	66 (30.7%)	
IV	345 (48.3%)	235 (47.1%)	110 (51.2%)	
Tumor size				0.385
<2cm/NOS	158 (22.1%)	106 (21.2%)	52 (24.2%)	
≥2cm	556 (77.9%)	393 (78.8%)	163 (75.8%)	
Nodes examined				0.755
<12	349 (48.9%)	242 (48.5%)	107 (49.8%)	
≥12	365 (51.1%)	257 (51.5%)	108 (50.2%)	
Nodes positive				0.504
Negative	365 (51.1%)	251 (50.3%)	114 (53.0%)	
Positive	349 (48.9%)	248 (49.7%)	101 (47.0%)	
LODDS				0.977
<1	336 (47.1%)	235 (47.1%)	101 (47.0%)	
≥1	378 (52.9%)	264 (52.9%)	114 (53.0%)	
Sugery				0.952
GTR/STR	486 (68.1%)	340 (68.1%)	146 (67.9%)	
Others	228 (31.9%)	159 (31.9%)	69 (32.1%)	
Radiation				0.975
No/Unknown	584 (81.8%)	408 (81.8%)	176 (81.9%)	
Yes	130 (18.2%)	91 (18.2%)	39 (18.1%)	
Chemotherapy				0.380
No/Unknown	263 (36.8%)	189 (37.9%)	74 (34.4%)	
Yes	451 (63.2%)	310 (62.1%)	141 (65.6%)	

### Feature selection and establishment of predictive model

**Figure 3:**
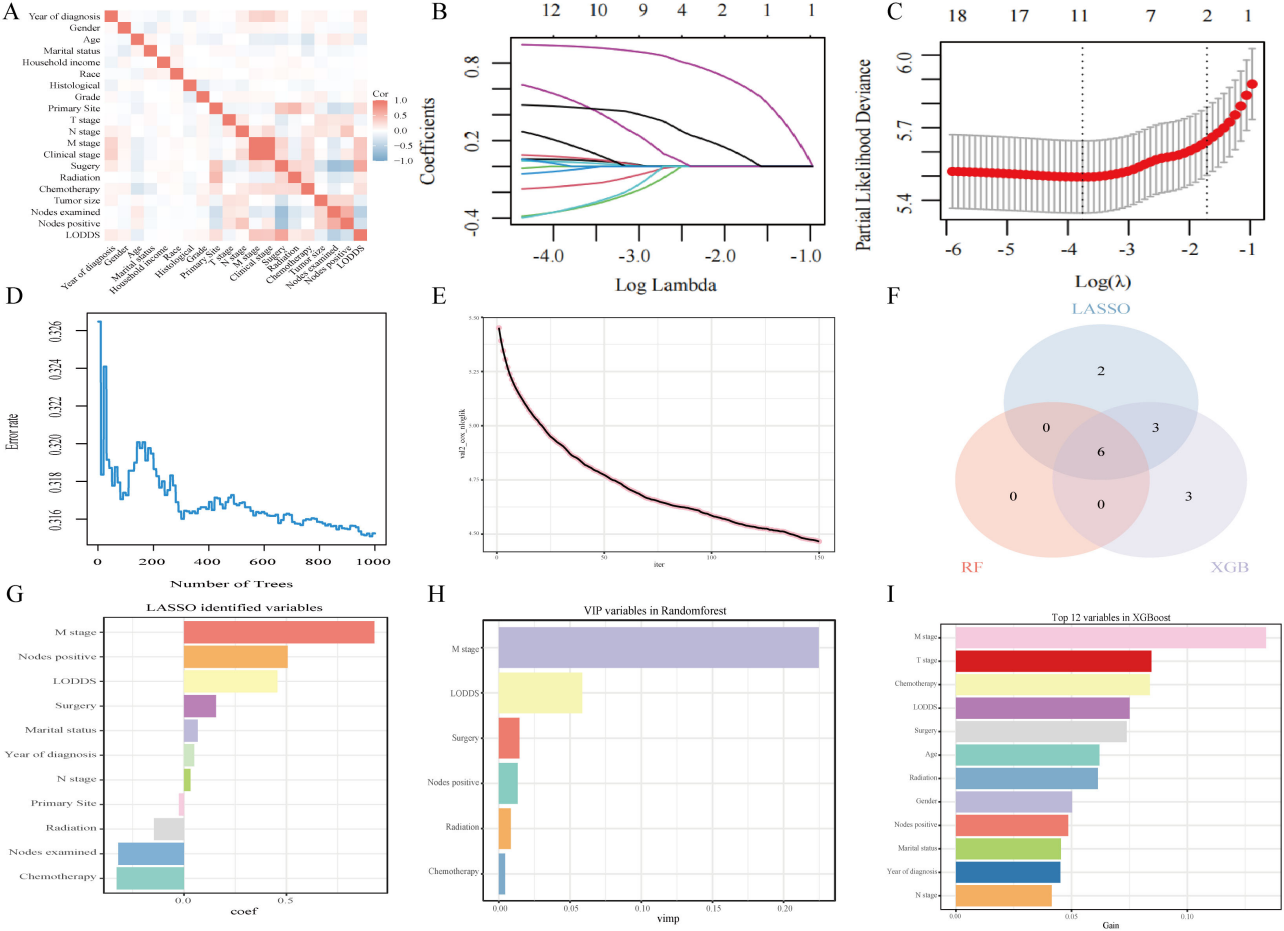
Analytical insights into OS using machine learning. **(A)** Correlation analysis among all incorporated variables. **(B)** Selection of tuning parameter (λ) in the LASSO model. **(C)** 10-fold cross-validation results. **(D)** OBB error rate derived from the Random Forest algorithm. **(E)** Learning curve plotted against the number of iterations. **(F)** Common variables identified across the three algorithms. **(G)** Variables identified through the LASSO model. **(H)** VIP variables selected via the Random Forest method. **(I)** Top 12 feature variables based on importance.

Prior to machine learning algorithm screening, potential collinearity among the examined parameters was assessed using Spearman correlation analysis, as illustrated in **Figure 3A**. **Supplementary Figures 2**–**4** show survival curves for each variable. In this investigation, we utilized three machine learning algorithms (LASSO, RF, and XGBoost) to screen variables for OS and CSS, effectively mitigating overfitting risks (15–17). LASSO regression was performed by minimizing the partial likelihood deviation, producing coefficient curves from a logarithmic (lambda) series, as depicted in **Figure 3B**. Utilizing 10-fold cross-validation, the algorithm identified critical clinical parameters that served as individual predictors in OS contexts (**Figure 3C**). The importance of each parameter within the LASSO analysis was further ranked to assess the predictive capability of each independent factor related to OS (**Figure 3G**). In the RF algorithm for OS, an increase in the number of random forests corresponded to a decline in the out-of-bag (OOB) error rate (**Figure 3D**). Subsequently, the Var.select function was used to isolate the VIP variables (**Figure 3H**). For the XGBoost algorithm, **Figure 3E** shows the learning curve relative to the iteration count and highlights the top 12 features (**Figure 3I**). The predictor variables for CSS underwent a similar filtration process (**Supplementary Figure 5**). In conclusion, six consistent parameters (M stage, LODDS, Nodes positive, Surgery, Radiotherapy, and Chemotherapy) pinpointed by all three algorithms (**Figure 3F**) were chosen as the ultimate predictor variables.

### Dynamic web version model deployment

**Figure 4:**
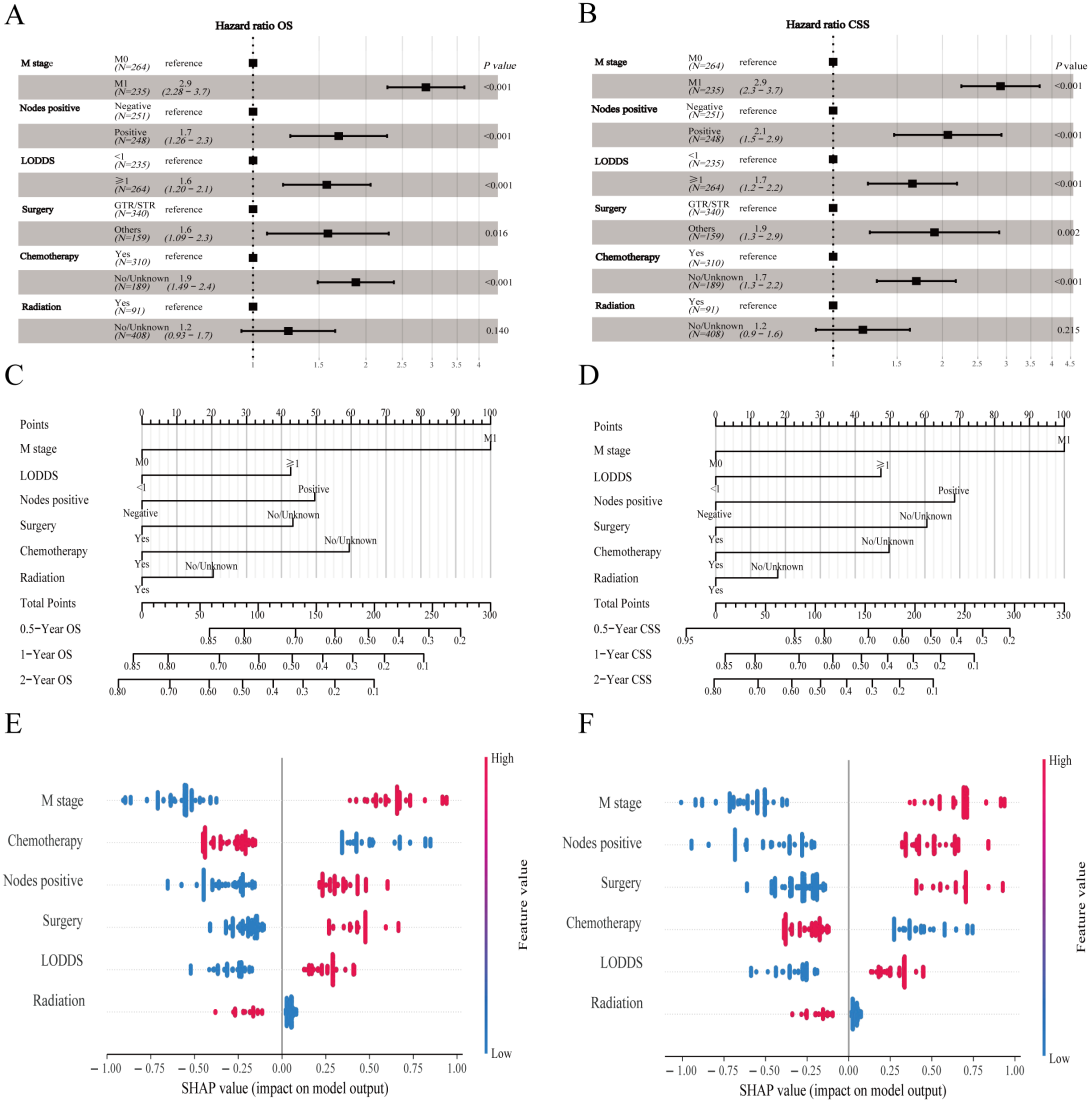
Machine learning models and predictive tools for patient survival. **(A)** Forest plot illustrating the machine learning model for OS. **(B)** Forest plot for the model on CSS in the training cohort. **(C)** Nomogram predicting 0.5-, 1-, and 2-year OS. **(D)** Nomogram for 0.5-, 1-, and 2-year CSS projections. **(E)** SHAP value visualization for Nomogram of OS. **(F)** SHAP value visualization for Nomogram of CSS.

The prediction model built based on six parameters is displayed with forest plots (**Figures 4A, B**) and visual nomograms (**Figures 4C, D**), and SHapley Additive exPlanation (SHAP) is used to implement the interpretability analysis of the model (**Figures 4E, F**). Each point represents a sample, and the color gradient from blue to red represents the size of the sample feature value. The vertical axis shows the importance ranking of features, as well as the correlation and distribution of each feature value with SHAP values. To aid researchers and clinicians in evaluating OS and CSS in patients with HCNEC, we introduced digital iterations of our model. These can be accessed at the following URLs: https://necr.shinyapps.io/NomoforHGNECRinOS/ and https://necr.shinyapps.io/NomoforHGNECRinCSS/.

### Internal and external multidimensional validation of models

**Figure 5:**
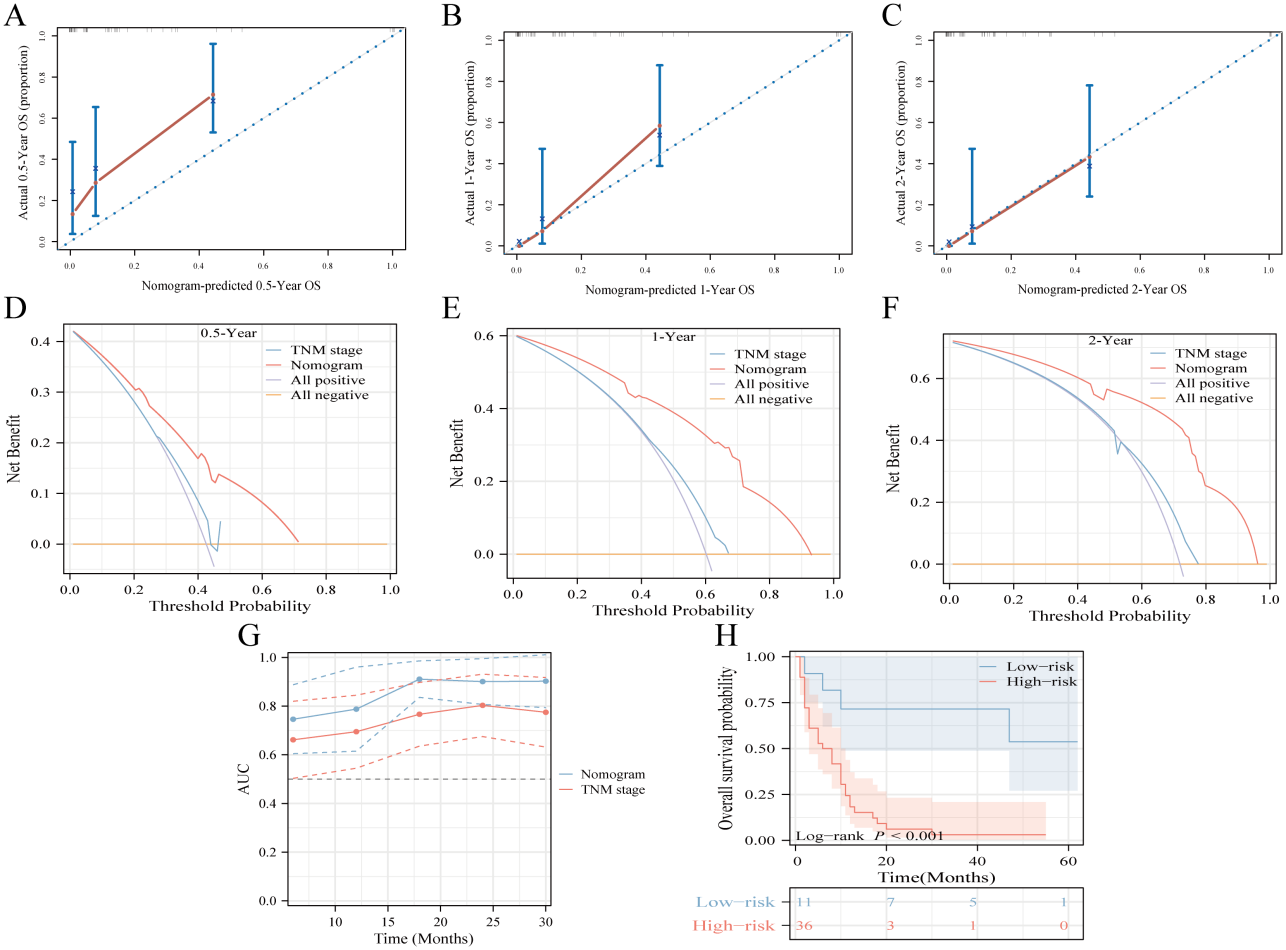
Multicenter external validation of predictive models. **(A-C)** Calibration curves forecasting OS at 0.5-year **(A)**, 1-year **(B)**, and 2-year **(C)** intervals. **(D-F)** Decision curve analysis for OS prediction at 0.5-year **(D)**, 1-year **(E)**, and 2-year **(F)** milestones, contrasting the model with the TNM-stage. **(G)** Time-dependent ROC curve comparison between the nomogram and TNM-stage system for OS. **(H)** Risk-stratification based on risk points derived from the model.

The proposed nomogram demonstrated significant proficiency in predicting OS at the 0.5-, 1-, and 2-year intervals. The C-index values for both the training (0.762) and validation (0.833) cohorts were 0.648 and 0.634, respectively, surpassing those of TNM stage. For 0.5-, 1-, and 2-year CSS predictions, our model outperformed the TNM staging system, achieving C-index scores of 0.761 and 0.665 for the training cohort and 0.831 and 0.652 for the validation cohort. Compared to TNM stage, our nomograms consistently presented a time-dependent AUC near 0.8, highlighting their superior predictive capability (**Supplementary Figure 6**). Calibration curves indicated a tight alignment between the predicted and observed survival rates, with the proposed models accurately predicting OS and CSS across all durations in both cohorts (**Supplementary Figures 7**, **8**). Decision curve analyses for the OS and CSS models validated their heightened clinical utility and predictive accuracy over the specified intervals, as evident from a broad spectrum of optimal threshold probabilities (**Supplementary Figure 9**). Moreover, in the external validation cohort, metrics such as the calibration curve, time-dependent AUC, DCA curve, and risk stratification analysis unequivocally showed the model’s robustness and superiority (**Figure 5**).

### Risk stratification and Sankey diagram based on the model

**Figure 6:**
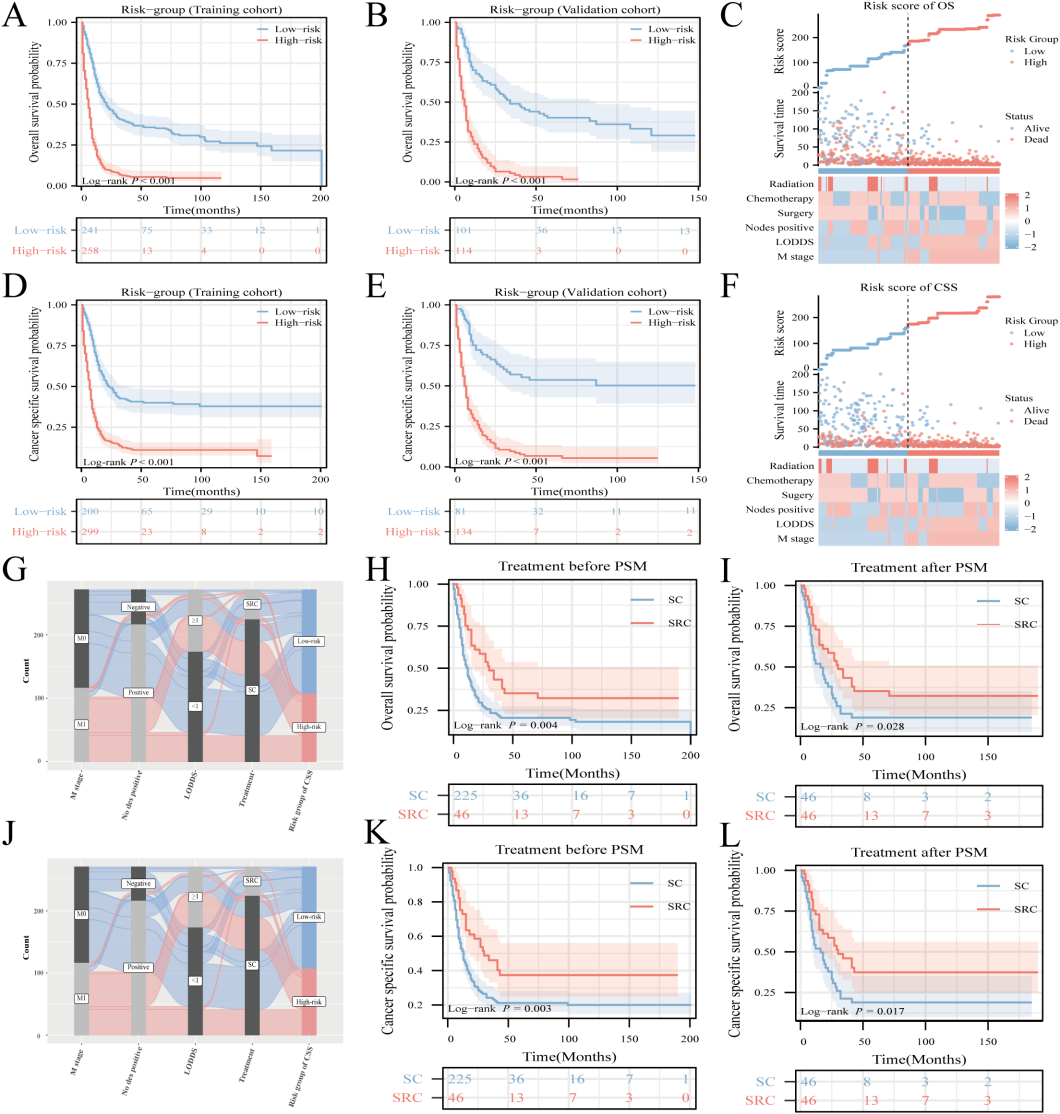
Risk-stratification, Sankey diagram based on the model and treatment strategy selection. Analysis of OS and CSS in HCNEC patients from the training cohort **(A, D)** juxtaposed against the validation cohort **(B, E)**. Clinicopathological feature distribution across varied risk groups for OS **(C)** and CSS **(F)**. The Sankey diagram delineates the relationship between predictor features and risk stratification for OS **(G)** and CSS **(J)**. Survival curves showcase the disparities between the two groups, both pre- and post-matching, for OS **(H, I)** and CSS **(K, L)**.

Notable differences in survival outcomes were observed between these risk groups (P<0.001), highlighting the utility of our nomogram and its stratification methodology (**Figures 6A, B, D, E**). To further illustrate the discrepancies in clinical characteristics among the designated risk categories for OS (**Figure 6C**) and CSS (**Figure 6F**), heat maps were utilized. A Sankey diagram, presented in **Figures 6G, J**, effectively captures the interplay between each factor and its consequent risk categorization. This visualization distinctly conveys how individual parameters contribute to the final risk classification, thereby enhancing the understanding of the model’s predictive capability.

### Optimal treatment strategy analysis

To investigate the impact of various treatments on patient prognosis, PSM analysis was used to minimize the effects of confounding variables (18, 19). The outcomes before and after PSM are presented in **Supplementary Table 3**. While literature on the survival benefits of radiotherapy for NEC remains scant, this study sought to contrast the results of triple therapy (encompassing surgery, radiotherapy, and chemotherapy) against those treatments combining surgery and chemotherapy, herein termed SC. Before the matching procedure, the triple-therapy regimen demonstrated superior OS and CSS outcomes compared to SC (**Figures 6H, K**). This advantage in OS and CSS for triple therapy remained evident after matching (**Figures 6I, L**).

### Genetic mutations and GO/KEGG analysis

**Figure 7:**
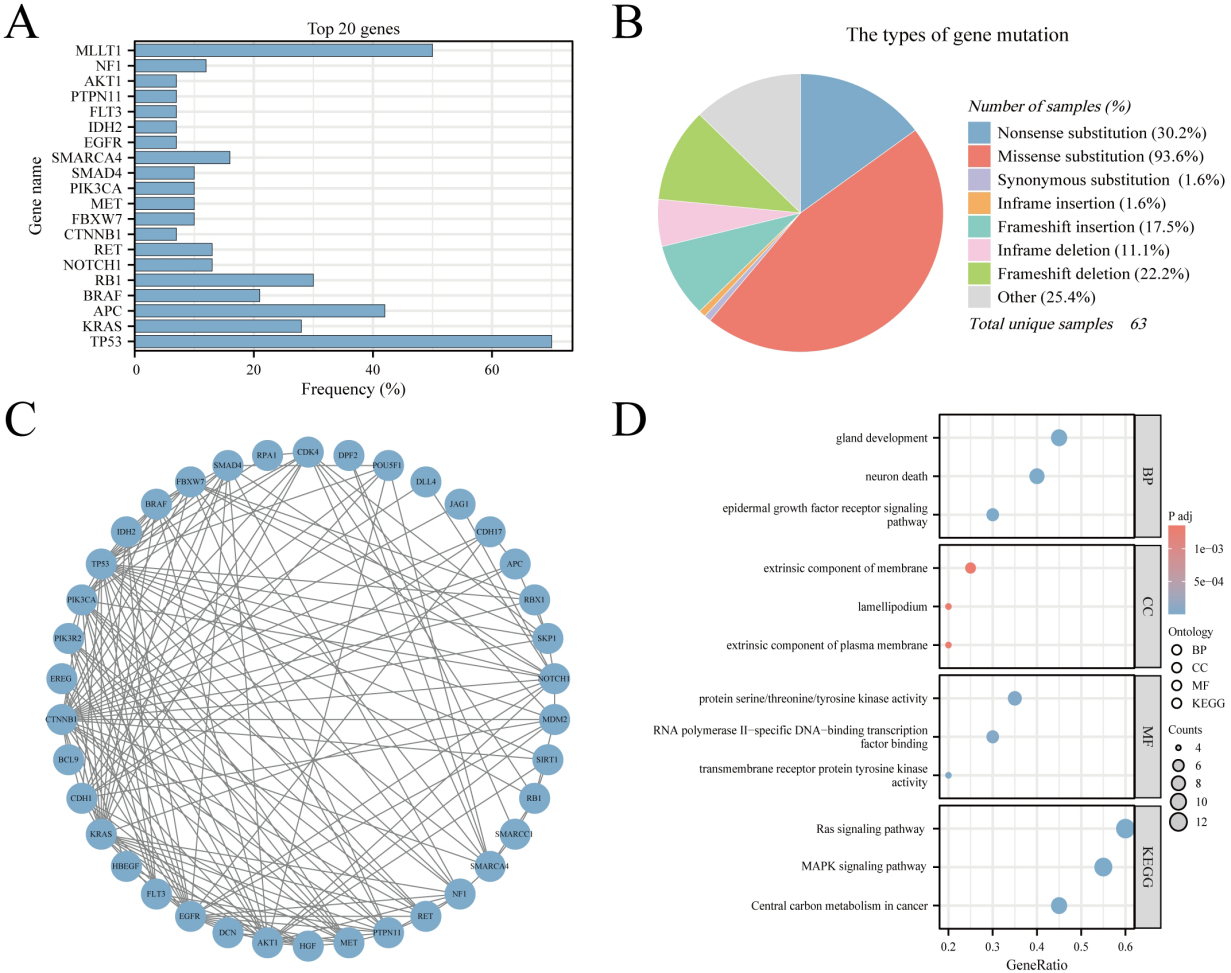
The Genomic Landscape, PPI and GO/KEGG analysis in CNEC. **(A)** The top 20 mutated genes. **(B)** An overview of the types of mutation observed. **(C)** PPI network of the top 20 mutated genes. **(D)** The GO/KEGG enrichment analysis of top 20 genes.

The CNEC genetic mutation data were extracted from COSMIC version GRCh38 COSMIC v99. In total, 55279 cases of colorectal tumors were evaluated for genetic mutations in the database. In the sub-tissue category, all colorectal sites were selected for data extraction. For histological selection, only CNEC cases were selected, and a final total of 63 cases were analyzed for genetic mutations. The top 20 genes that were mutated in CNEC were TP53 70% (in all samples tested = 43), KRAS 28% (96), APC 42% (43), BRAF 21% (81), RB1 30% (30), NOTCH1 13% (30), RET 13% (30), CTNNB1 7% (41), FBXW7 10% (30), MET 10% (30), PIK3CA 10% (30), SMAD4 10% (30), SMARCA4 16% (19), EGFR 7% (30), IDH2 7% (30), FLT3 7% (30), PTPN11 7% (30), AKT1 7% (30), NF1 12% (17), and MLLT1 50% (4) (**Figure 7A**). RT-QPCR results based on clinical samples also showed that the expression of TP53, KRAS, APC, BRAF and RB1 genes increased significantly in HCNEC tissues (**Supplementary Figure 10**). An overview of the mutation types and PPI network are shown in **Figures 7B, C**. We performed GO and KEGG analyses of these genes (20). Biological process analysis showed that the top 20 genes were enriched in gland development, extrinsic component of membrane, protein kinase activity, EGFR and Ras signaling pathways (**Figure 7D**).

## Discussion

HCNEC, a rare and aggressive tumor, exhibits traits akin to small cell lung cancer, particularly in terms of pronounced invasion and metastasis (8, 21). Most HCNEC patients are diagnosed at advanced stages or when distant metastasis has already manifested, resulting in a bleak prognosis (22, 23). Owing to the rarity of HCNEC cases, comprehensive research in this area poses considerable challenges. The SEER database, a respected source of U.S. cancer statistics, is invaluable for studying rare tumors (24–27). From an in-depth analysis, we extracted a substantial sample of HCNEC data from SEER (n=714) and employed machine learning techniques to discern six pivotal clinical factors that correlated with OS and CSS. Additionally, we used the COSMIC database to analyze the genomic variation characteristics of CNEC. To our knowledge, this study represents a pioneering effort to leverage SEER data and COSMIC data to establish specific survival prediction models and gene mutation landscapes, respectively. To enhance the practical relevance of our conclusions, we integrated web-based prognostic tools and introduced SHAP visual representation to optimize risk-informed clinical decision-making.

Our epidemiological survey demonstrated a noticeable increase in the incidence of CNEC over the past two decades. Given the scarcity of early research and recent advancements in endoscopic diagnostic and therapeutic approaches, the actual incidence of NEC, especially within the gastrointestinal domain, is anticipated to increase (28, 29). Consequently, it is imperative to prioritize and enhance NEC related management in the future. Evidence suggests that HCNEC possess notable invasive and metastatic capacity (30, 31). In alignment with this, our analysis revealed a significant number of patients with newly diagnosed metastasis exhibiting an unfavorable prognosis (M1 = 345, 48.3%). It has been established that tumors within the digestive system frequently metastasize to lymph nodes, often resulting in a poor prognosis (32–35). Prior research has underscored the correlation between prognosis and parameters such as the number of dissected lymph nodes, number of positive lymph nodes, and ratio of positive lymph nodes in patients with colorectal cancer (36, 37). In our investigation, the LODDS algorithm, recognized for its precision, was employed to elucidate the association between lymph nodes and HCNEC (38, 39). These findings substantiated that both positive lymph nodes and LODDS > 1 serve as reliable prognostic markers, offering advantages over traditional N staging. Moreover, these findings maintained consistent validation even within the constraints of our limited external dataset.

According to the National Comprehensive Cancer Network (NCCN) Neuroendocrine Tumor Guidelines, resectable NEC should undergo surgery and systemic chemotherapy, optionally complemented by local radiotherapy, paralleling the treatment approach for small cell lung cancer (13). Surgery continues to be the primary diagnostic and therapeutic intervention for patients (12). A thorough examination of the database’s surgical definitions revealed significant survival benefits for patients who underwent gross total resection or subtotal resection (GTR/STR). Numerous prior studies have convincingly demonstrated that patients with gastroenteropancreatic neuroendocrine tumors who receive R0 surgical resection of the primary tumor experience substantial survival advantages (40, 41). In conclusion, R0/R1 resection should always be regarded as the first-line treatment when achievable. For cases of locally advanced, unresectable, or metastatic CNEC, frontline therapy involves a combination of cisplatin/carboplatin and etoposide (EP regimen) (14, 42). Several retrospective studies with limited sample sizes have reported an objective response rate (ORR) for this regimen ranging between 30% and 70%, with a median OS spanning 11-19 months (43, 44). In addition, a phase 2 clinical trial from a multi-center randomized controlled trial showed that after the failure of the EP regimen, the FOLFIRI regimen (irinotecan, leucovorin, and fluorouracil) can be regarded as the standard second-line treatment for patients with gastroenteropancreatic neuroendocrine cancer (45). Our study corroborates the significance of chemotherapy as a pivotal factor for patient prognosis. The adoption of radiation therapy in HCNEC remains infrequent (n=130, 18.2%), with little previous research. Machine learning algorithm outcomes denote radiotherapy as a consequential variable influencing HCNEC prognosis, although its distinction in the multi-factor Cox forest plot remains unclear (P=0.140 in OS, P=0.215 in CSS). Given the paucity of prior investigations and indeterminate outcomes, we leveraged the PSM technique to equalize confounding elements and juxtaposed survival rates of SRC (amalgamating surgery, radiotherapy, and chemotherapy) and SC (surgery combined with chemotherapy) patient groups. The findings revealed that the tri-modal treatment yielded pronounced survival advantages both pre- and post-matching. The pivotal role of RT was further reinforced using our external dataset (**Supplementary Figure 4O**). This highlights the importance of integrating local radiotherapy with combination treatments and could offer pivotal insights for ensuing clinical prospective studies. In summary, addressing aggressive malignancies, such as HCNEC, mandates a multifaceted therapeutic strategy. Anticipation builds for future clinical trials to elucidate and affirm these progressive methods.

The pathogenesis of HCNEC remains unclear, and there is a notable absence of targeted therapeutic drugs currently available in clinical practice. Multiple previous studies on neuroendocrine tumors have confirmed that this type of disease is related to multiple gene mutations/deletions, such as TP53, RB1, CTNNB1, NF1, etc (46–48). Our mutation gene analysis results also showed that the most common mutations in CNEC were TP53, KRAS, APC, BRAF, and RB1, and were verified from independent clinical samples. As the most common tumor suppressor gene, TP53 mutations or functional inactivation are associated with poor prognosis in pan-cancers, including colorectal tumor (49). The GO/KEGG analysis based on the top 20 mutated gene sets also revealed that the biological properties of CNEC are related to EGFR, RAS, MAPK signaling pathway. Multiple studies have proven that EGFR mutations may be involved in the process of tumors becoming neuroendocrine. Targeting signaling pathways such as RAS and MAPK can inhibit the growth of neuroendocrine tumors (50–52). Consequently, conducting an in-depth genomic analysis of high-grade CNEC patients with poor prognoses is essential, as it may reveal potential therapeutic targets for this disease.

In this study, machine learning algorithms were employed to develop a model for evaluating survival risks. Rigorous validation demonstrated the model’s exceptional precision, indicating that it is more effective than the prevailing TNM staging system. However, there are some limitations to our research. Owing to the retrospective nature of the study, patients not listed in the SEER registry were excluded, potentially leading to sampling bias. Additionally, the SEER database does not provide comprehensive information on crucial clinical parameters, such as performance status, distinct chemotherapy protocols, number of treatment cycles, radiation dosage, and subsequent therapeutic lines, which may cause survival bias. Furthermore, the lack of indicators, such as disease progression-free survival and recurrence survival, within the SEER database might restrict the model’s widespread applicability. Genomics analysis needs to be more in-depth, such as exploration of epigenetic changes and functional mechanisms.

## Conclusions

In conclusion, we systematically analyzed patient data from the SEER database from 2000 to 2019 and the genetic mutation characteristics of the patients in the COSMIC database. From this analysis, we identified several clinical factors that independently influenced OS and CSS in patients with HCNEC and mapped the genetic mutation landscape. The developed prediction model, notable for its precision, presents a potential instrument for tackling prevailing clinical hurdles. Additionally, analysis based on mutational genomics will facilitate future research on molecular targeted drugs.

## Data Availability

The original contributions presented in the study are included in the article/**Supplementary Material**. Further inquiries can be directed to the corresponding authors.
