# Supplementary material for: Machine learning based predictive model and genetic mutation landscape for high-grade colorectal neuroendocrine carcinoma: a SEER database analysis with external validation
Source: Front Oncol. 2025 Jan 29;15:1509170. doi: 10.3389/fonc.2025.1509170 (PMC11813786; doi:10.3389/fonc.2025.1509170)
Supplement: Supplementary file 1 [file DataSheet1.docx]

Supplementary Material


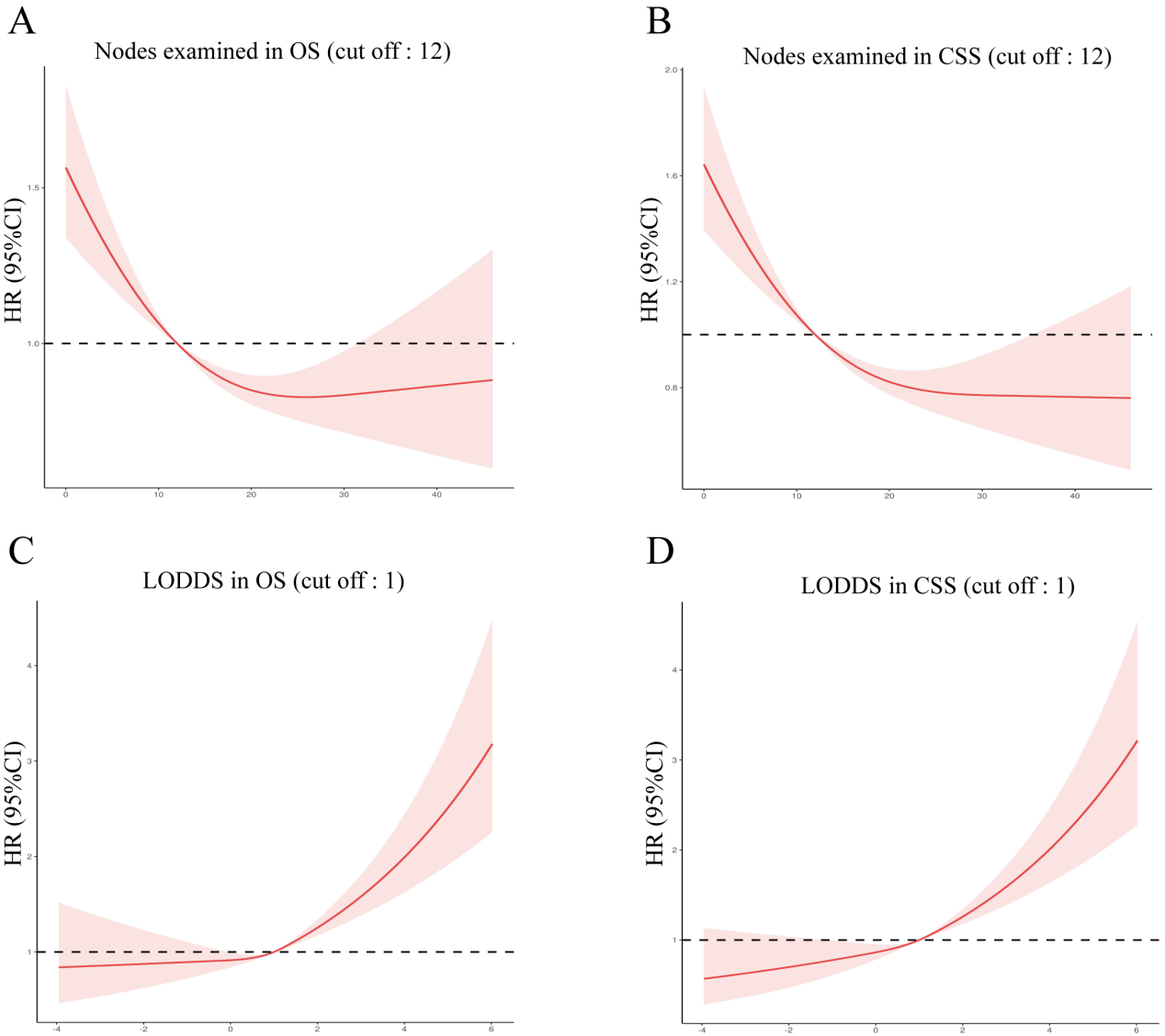


Supplementary Figure 1. RCS determines the optimal cutoff value for lymph node detection number (A,B) and LODDS (C,D) in OS and CSS.


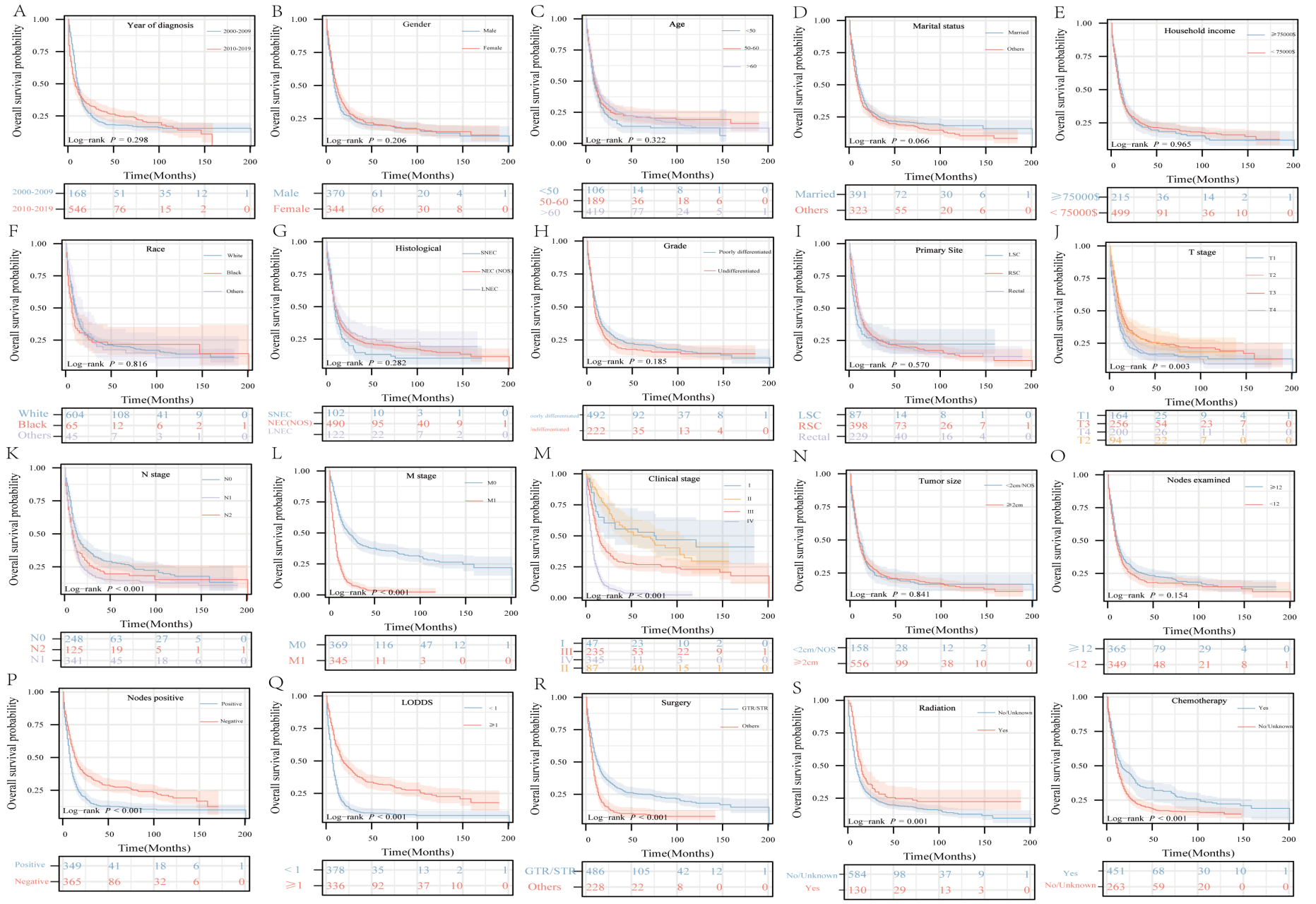


Supplementary Figure 2. The survival curve for each variable in OS.


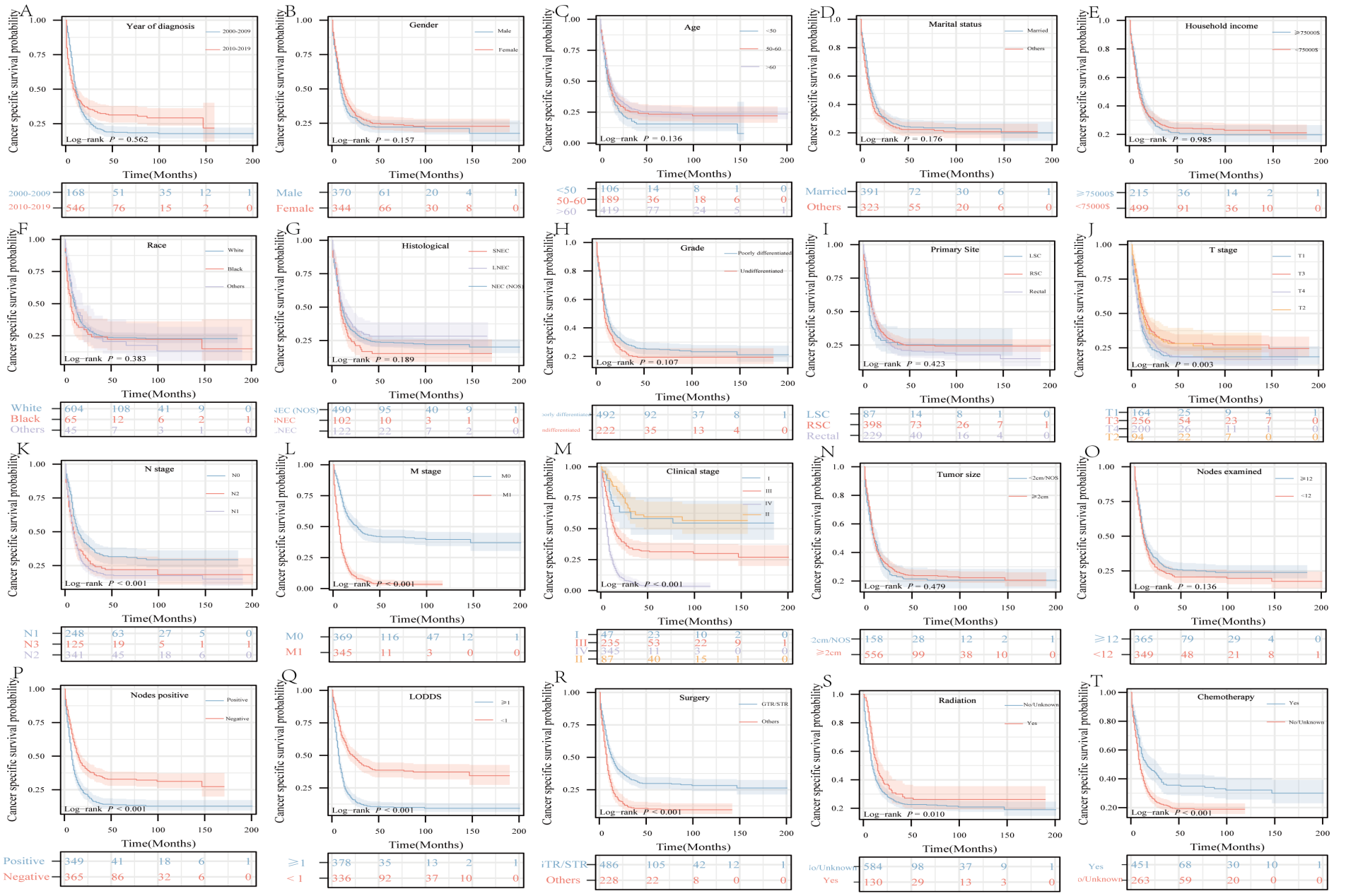


Supplementary Figure 3. The survival curve for each variable in CSS.


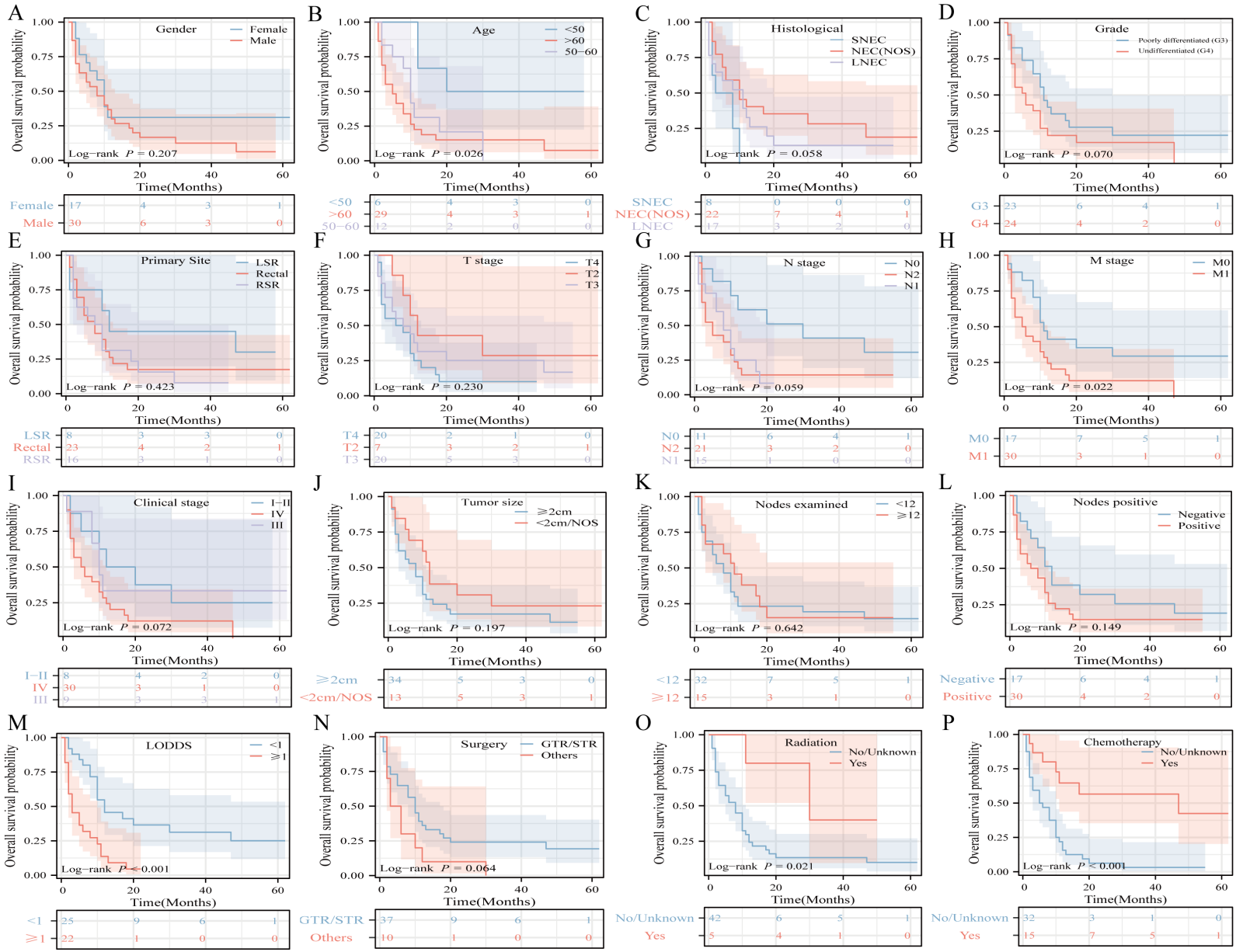


Supplementary Figure 4. The survival curves of each variable in the external validation cohort.


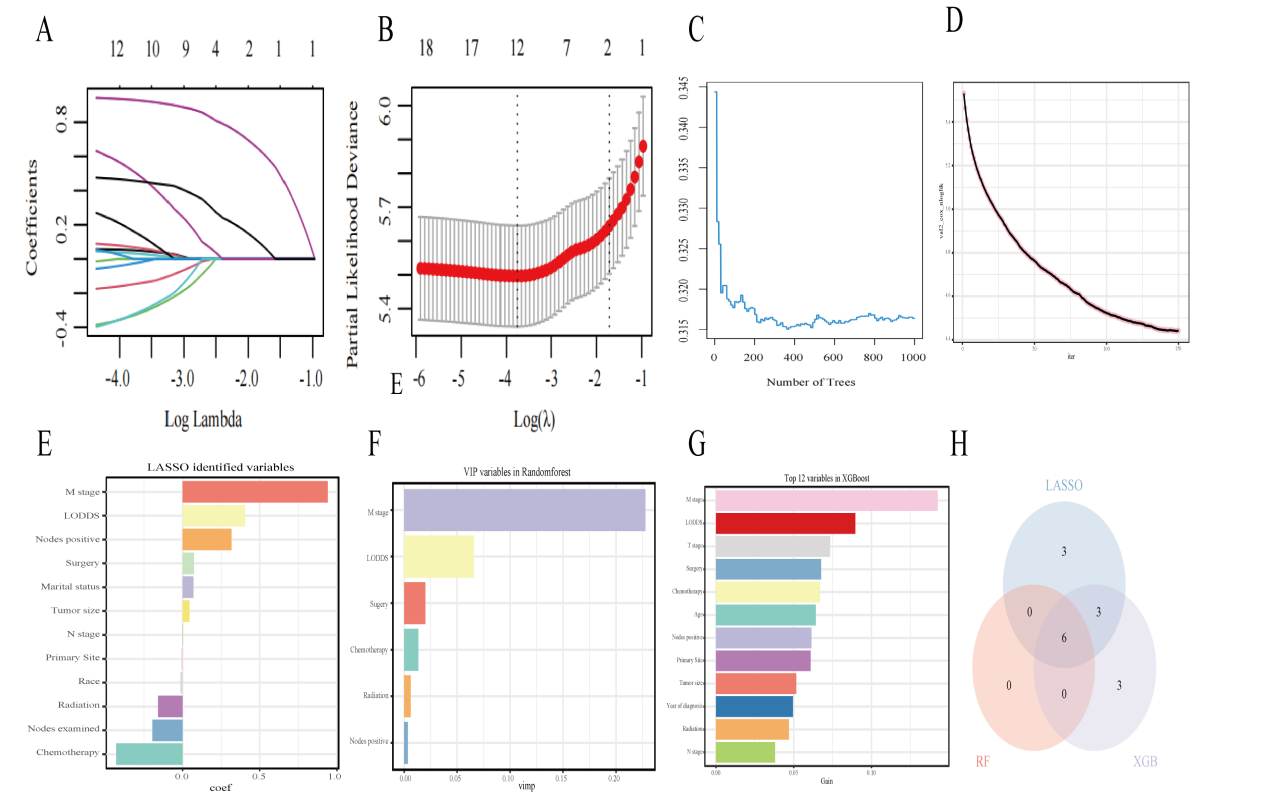
Supplementary Figure 5.Machine learning model in CSS. Selection of tuning parameter (λ) for the LASSO model (A), 10-fold cross-validation (B) and identified variables (E); The OBB error rate of Random forest (C), and selected VIP variables (F); Learning curve as a function of number of iterations (D) and Top 12 important feature variables (G). Intersection variables of the three algorithms (H).


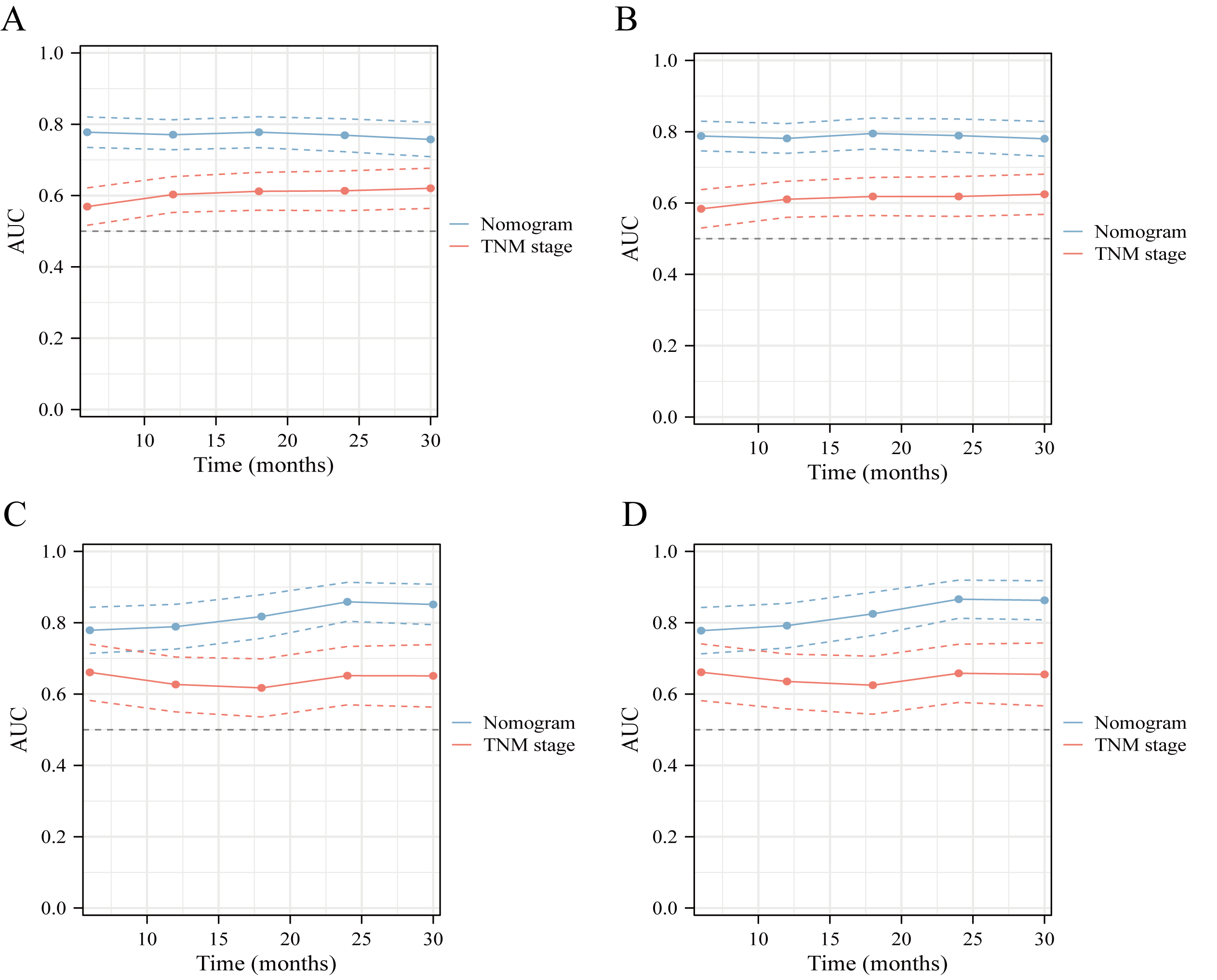


Supplementary Figure 6**.** Comparison of the area under the ROC curve for time-dependent OS and CSS duration in the nomogram and TNM-stage system prediction training cohort (A, C), internal validation cohort (B, D).


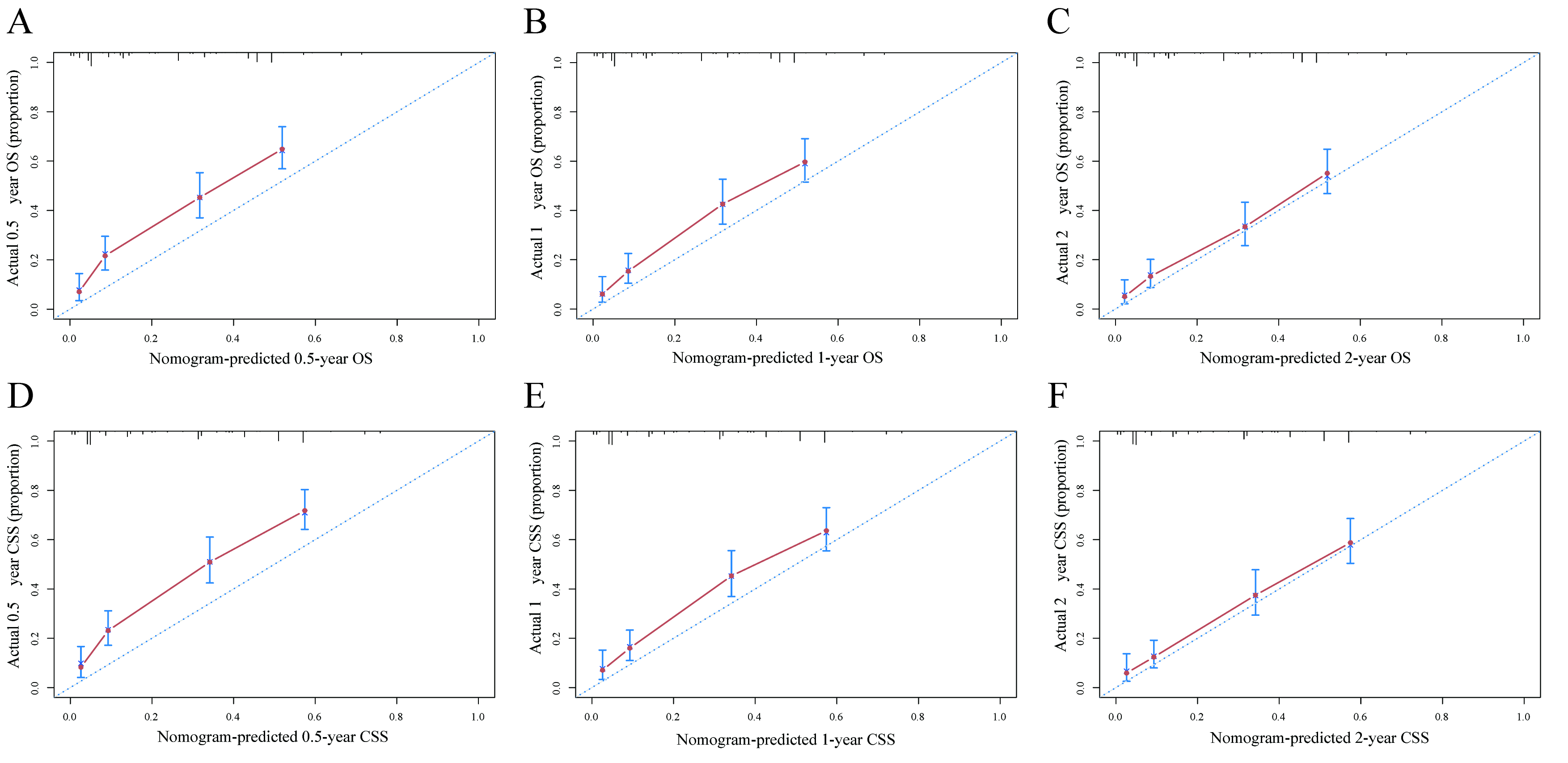


Supplementary Figure 7. The calibration curves predicting 0.5-year (A,D), 1-year (B,E), and 2-year (C,F) OS in training group and validation group.


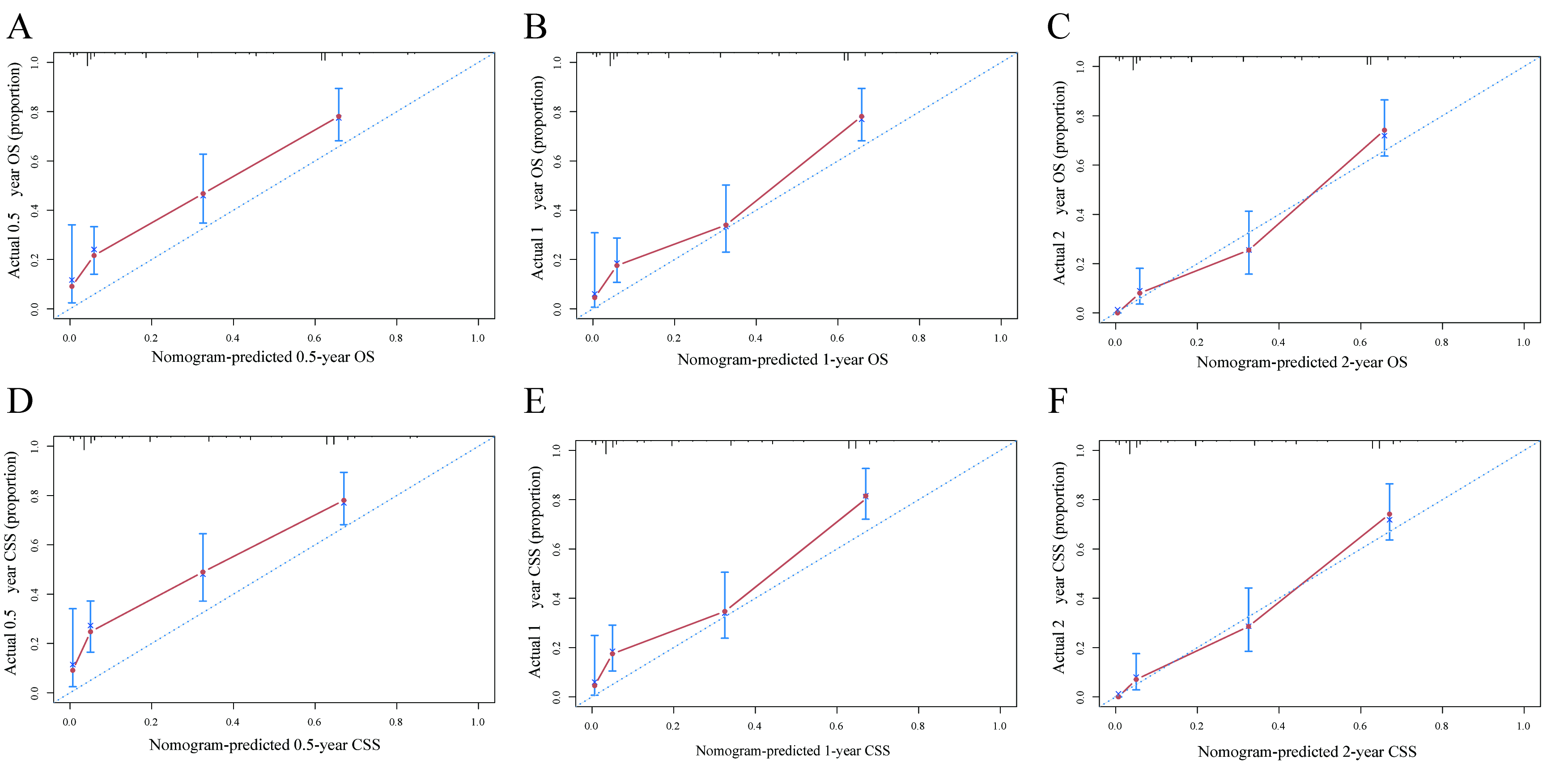


Supplementary Figure 8. The calibration curves predicting 0.5-year (A,D), 1-year (B,E), and 2-year (C,F) CSS in training group and validation group.


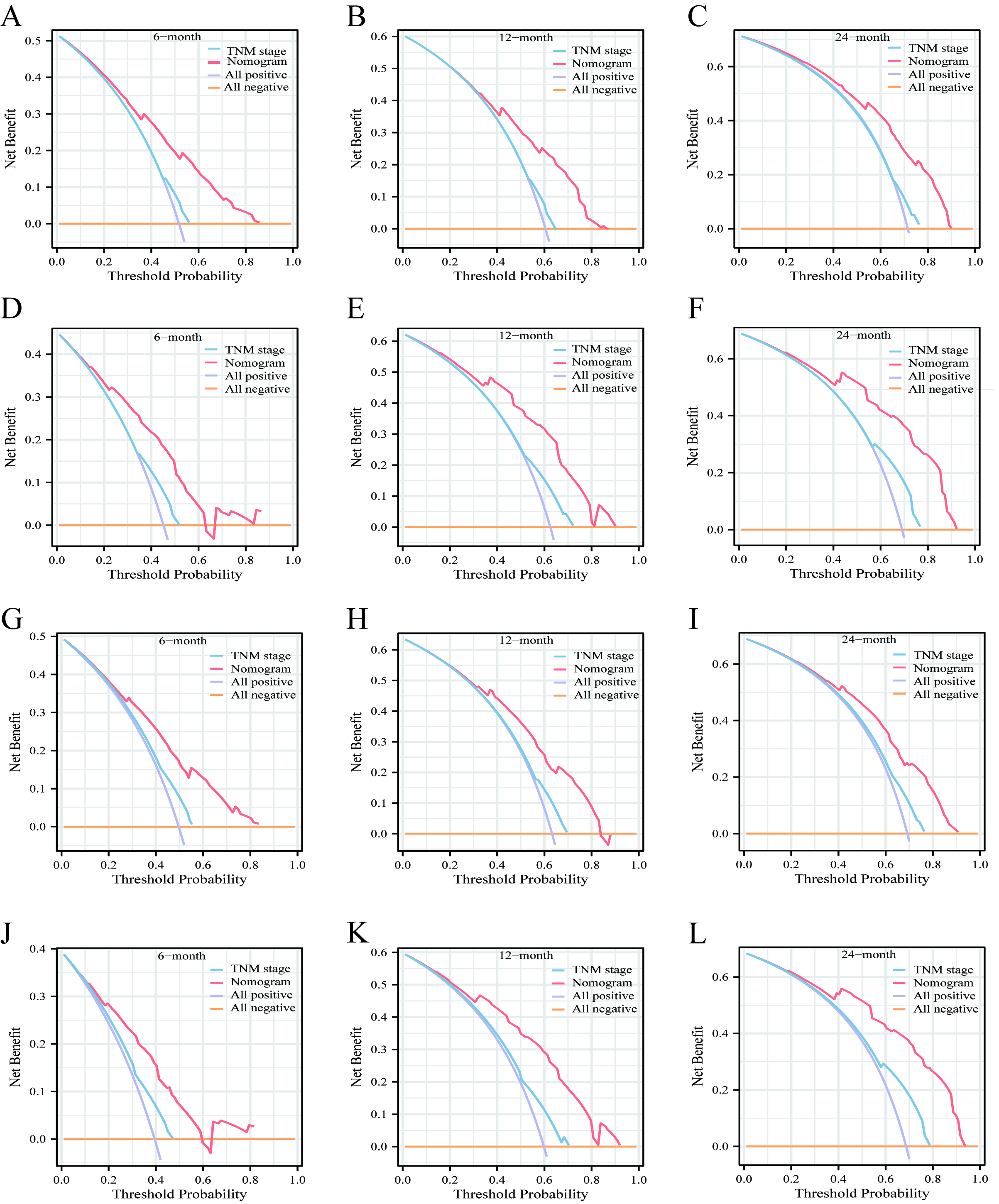


Supplementary Figure 9. The nomogram and the TNM-stage of the Decision curve analysis in the prediction of OS at the 0.5-year (A,D), 1-year (B,E) and 2-year (C,F) point in the training and validation groups. The prediction of CSS at the 0.5-year (G,J), 1-year (H,K) and 2-year (I,L) point in the training and validation groups.


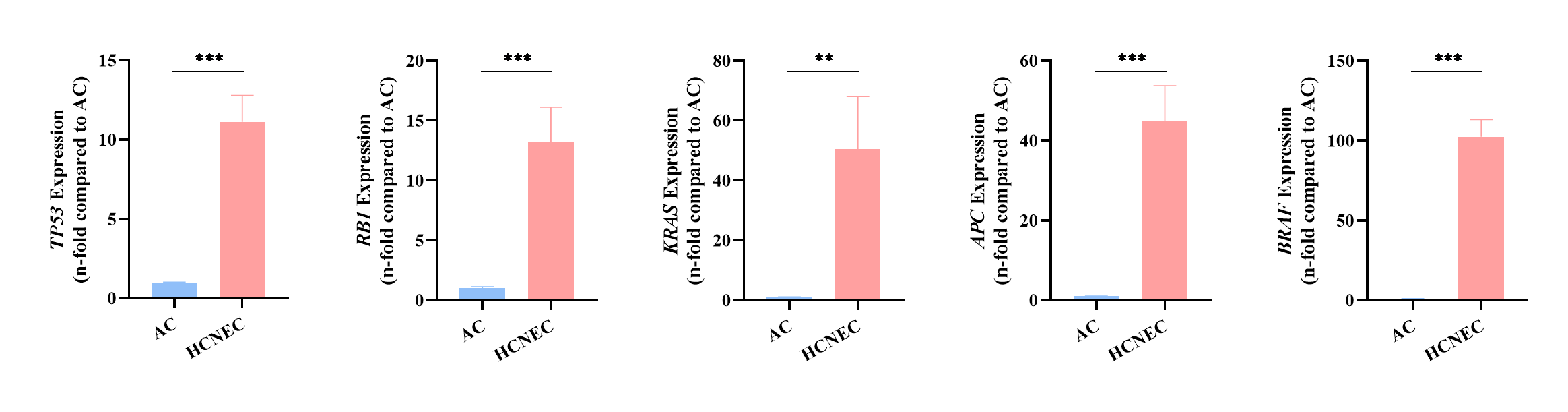


Supplementary Figure 10. Comparison of Top 5 mutated genes (TP53, KRAS, APC, BRAF and RB1) mRNA expression levels obtained from HCNEC and normal tissues using RT-qPCR.

Supplementary Table 1. Primers for quantitative RT-PCR

| **Gene** | **Forward Primer Sequence (5'to3')** | **Reverse Primer Sequence (5'to3')** |
| --- | --- | --- |
| **MLLT1** | GAGGGGTTCACTCACGACTG | GGCATGATGAAGCCAGCGTA |
| **APC** | AAGCATGAAACCGGCTCACAT | CATTCGTGTAGTTGAACCCTGA |
| **RB1** | CTCTCGTCAGGCTTGAGTTTG | GACATCTCATCTAGGTCAACTGC |
| **KRAS** | ACAGAGAGTGGAGGATGCTTT | TTTCACACAGCCAGGAGTCTT |
| **BRAF** | AATACACCAGCAAGCTAGATGC | AATCAGTTCCGTTCCCCAGAG |
| **TP53** | CAGCACATGACGGAGGTTGT | TCATCCAAATACTCCACACGC |

Supplementary Table 2**.** Characteristics of the HCNEC patients in the external validation group.

| **Characteristics** | **External validation group in Guizhou (n=26)**  **no.(%)** | **External validation group in Chongqing (n=21)**  **no.(%)** |
| --- | --- | --- |
| **Median follow-up time**（**Months, Range**） | 50(16.8-83.2) | 45(23.1-66.9) |
| **Median overall survival time**（**Months, 95%CI**） | 8(3.3-12.7) | 10(5.5-14.6) |
| **Gender** |  |  |
| Male | 16 (61.5%) | 7(33.3%) |
| Female | 10 (38.5%) | 14(66.7%) |
| **Age** |  |  |
| <50 | 3 (11.5%) | 3(14.3%) |
| 50-60 | 5 (19.2%) | 7(33.3%) |
| >60 | 18 (69.3%) | 11(52.4%) |
| **Marital status** |  |  |
| Married | 26 (100.0%) | 21 (100.0%) |
| Others | 0(0.0%) | 0(0.0%) |
| **Race** |  |  |
| White | 0(0.0%) | 0(0.0%) |
| Black | 0(0.0%) | 0(0.0%) |
| Others | 26 (100.0%) | 21(100.0%) |
| **Histological** |  |  |
| SNEC | 4 (15.4%) | 4(19.1%) |
| LNEC | 10 (38.5%) | 7(33.3%) |
| NEC (NOS) | 12 (46.1%) | 10(47.6%) |
| **Grade** |  |  |
| Poorly differentiated | 17 (65.4%) | 6(28.6%) |
| Undifferentiated | 9 (34.6%) | 15(71.4%) |
| **Primary Site** |  |  |
| LSC | 6 (23.1%) | 2(9.5%) |
| RSC | 5 (19.2%) | 11(52.4%) |
| Rectal | 15 (57.7%) | 8(38.1%) |
| **T stage** |  |  |
| T1 | 0(0.0%) | 0(0.0%) |
| T2 | 5 (19.2%) | 2(9.5%) |
| T3 | 10 (38.5%) | 10(47.6%) |
| T4 | 11 (42.3% | 9(42.9%) |
| **N stage** |  |  |
| N0 | 6 (23.1%) | 5(23.8%) |
| N1 | 7 (26.9%) | 8(38.1%) |
| N2 | 13 (50.0%) | 8(38.1%) |
| **M stage** |  |  |
| M0 | 8 (30.8%) | 9(42.9%) |
| M1 | 18 (69.2%) | 12(57.1%) |
| **Clinical stage** |  |  |
| Ⅰ | 1 (3.8%) | 0(0.0%) |
| Ⅱ | 3 (11.5%) | 4(19.1%) |
| Ⅲ | 4 (15.4%) | 5(23.8%) |
| Ⅳ | 18 (69.3%) | 12(57.1%) |
| **Tumor size** |  |  |
| <2cm/NOS | 6 (23.1%) | 7(33.3%) |
| ≥2cm | 20 (76.9%) | 14(66.7%) |
| **Nodes examined** |  |  |
| <12 | 19 (73.1%) | 13(61.9%) |
| ≥12 | 7 (26.9%) | 8(38.1%) |
| **Nodes positive** |  |  |
| Negative | 12 (46.2%) | 5(23.8%) |
| Positive | 14 (53.8%) | 16(76.2%) |
| **LODDS** |  |  |
| <1 | 14 (53.8%) | 11(52.4%) |
| ≥1 | 12 (46.2%) | 10(47.6%) |
| **Sugery** |  |  |
| GTR/STR | 9 (34.6%) | 3(14.3%) |
| Others | 17 (65.4%) | 18(85.7%) |
| **Radiation** |  |  |
| No/Unknown | 23 (88.5%) | 19(90.5%) |
| Yes | 3 (11.5%) | 2(9.5%) |
| **Chemotherapy** |  |  |
| No/Unknown | 18 (69.2%) | 14(66.7%) |
| Yes | 8 (30.8%) | 7 (33.3%) |

Supplementary Table 3. Propensity-matched score analysis of triple-therapy (SRC) with surgery plus chemotherapy (SC) in HCNEC.

| **Characteristics** | Unmatched | | *P value* | Matched | | *P value* |
| --- | --- | --- | --- | --- | --- | --- |
|  | SC | SRC |  | SC | SRC |  |
|  | N=225 | N=46 |  | N=46 | N=46 |  |
| **Year of diagnosis** |  |  | 0.317 |  |  | 0.497 |
| 2000-2009 | 59 (26.2%) | 16 (34.8%) |  | 12 (26.1%) | 16 (34.8%) |  |
| 2010-2019 | 166 (73.8%) | 30 (65.2%) |  | 34 (73.9%) | 30 (65.2%) |  |
| **Gender** |  |  | 0.262 |  |  | 0.677 |
| Male | 126 (56%) | 21 (45.7%) |  | 24 (52.2%) | 21 (45.7%) |  |
| Female | 99 (44%) | 25 (54.3%) |  | 22 (47.8%) | 25 (54.3%) |  |
| **Age** |  |  | 0.678 |  |  | 0.732 |
| <50 | 35 (15.6%) | 5 (10.9%) |  | 3 (6.5%) | 5 (10.9%) |  |
| 50-60 | 70 (31.1%) | 14 (30.4%) |  | 13 (28.3%) | 14 (30.4%) |  |
| >60 | 120 (53.3%) | 27 (58.7%) |  | 30 (65.2%) | 27 (58.7%) |  |
| **Marital status** |  |  | 0.115 |  |  | >0.999 |
| Married | 148 (65.8%) | 24 (52.2%) |  | 24 (52.2%) | 24 (52.2%) |  |
| Others | 77 (34.2%) | 22 (47.8%) |  | 22 (47.8%) | 22 (47.8%) |  |
| **Household income** |  |  | 0.838 |  |  | >0.999 |
| ≥75000$ | 67 (29.8%) | 15 (32.6%) |  | 14 (30.4%) | 15 (32.6%) |  |
| <75000$ | 158 (70.2%) | 31 (67.4%) |  | 32 (69.6%) | 31 (67.4%) |  |
| **Race** |  |  | 0.462 |  |  | >0.999 |
| White | 196 (87.1%) | 38 (82.6%) |  | 37 (80.4%) | 38 (82.6%) |  |
| Black | 16 (7.1%) | 3 (6.5%) |  | 3 (6.5%) | 3 (6.5%) |  |
| Others | 13 (5.8%) | 5 (10.9%) |  | 6 (13%) | 5 (10.9%) |  |
| **Histological** |  |  | 0.258 |  |  | 0.711 |
| SNECR | 24 (10.7%) | 6 (13%) |  | 5 (10.9%) | 6 (13%) |  |
| LNECR | 60 (26.7%) | 7 (15.2%) |  | 10 (21.7%) | 7 (15.2%) |  |
| NECR (NOS) | 141 (62.7%) | 33 (71.7%) |  | 31 (67.4%) | 33 (71.7%) |  |
| **Grade** |  |  | 0.985 |  |  | 0.668 |
| Poorly differentiated | 150 (66.7%) | 30 (65.2%) |  | 27 (58.7%) | 30 (65.2%) |  |
| Undifferentiated | 75 (33.3%) | 16 (34.8%) |  | 19 (41.3%) | 16 (34.8%) |  |
| **Primary Site** |  |  | <0.001 |  |  | 0.145 |
| LSC | 36 (16%) | 0 (0%) |  | 0 (0%) | 0 (0%) |  |
| RSC | 162 (72%) | 13 (28.3%) |  | 26 (56.5%) | 18(39.1%) |  |
| Rectal | 27 (12%) | 33 (71.7%) |  | 20 (43.5%) | 28(60.9%) |  |
| **T stage** |  |  | 0.003 |  |  | 0.553 |
| T1 | 41 (18.2%) | 6 (13%) |  | 10 (21.7%) | 6 (13%) |  |
| T2 | 20 (8.9%) | 4 (8.7%) |  | 6 (13%) | 4 (8.7%) |  |
| T3 | 79 (35.1%) | 29 (63%) |  | 23 (50%) | 29 (63%) |  |
| T4 | 85 (37.8%) | 7 (15.2%) |  | 7 (15.2%) | 7 (15.2%) |  |
| **N stage** |  |  | 0.189 |  |  | 0.764 |
| N0 | 48 (21.3%) | 13 (28.3%) |  | 10 (21.7%) | 13 (28.3%) |  |
| N1 | 121 (53.8%) | 27 (58.7%) |  | 29 (63%) | 27 (58.7%) |  |
| N2 | 56 (24.9%) | 6 (13%) |  | 7 (15.2%) | 6 (13%) |  |
| **M stage** |  |  | 0.006 |  |  | 0.257 |
| M0 | 119 (52.9%) | 35 (76.1%) |  | 29 (63%) | 35 (76.1%) |  |
| M1 | 106 (47.1%) | 11 (23.9%) |  | 17 (37%) | 11 (23.9%) |  |
| **Clinical stage** |  |  | <0.001 |  |  | 0.479 |
| Ⅰ | 5 (2.2%) | 3 (6.5%) |  | 3 (6.5%) | 3 (6.5%) |  |
| Ⅱ | 12 (5.3%) | 9 (19.6%) |  | 5 (10.9%) | 9 (19.6%) |  |
| Ⅲ | 102 (45.3%) | 23 (50%) |  | 21 (45.7%) | 23 (50%) |  |
| Ⅳ | 106 (47.1%) | 11 (23.9%) |  | 17 (37%) | 11 (23.9%) |  |
| **Tumorsize** |  |  | 0.041 |  |  | 0.591 |
| <2cm/NOS | 22 (9.8%) | 10 (21.7%) |  | 7 (15.2%) | 10 (21.7%) |  |
| ≥2cm | 203 (90.2%) | 36 (78.3%) |  | 39 (84.8%) | 36 (78.3%) |  |
| **Nodes examined** |  |  | 0.006 |  |  | 0.668 |
| <12 | 47 (20.9%) | 19 (41.3%) |  | 16 (34.8%) | 19 (41.3%) |  |
| ≥12 | 178 (79.1%) | 27 (58.7%) |  | 30 (65.2%) | 27 (58.7%) |  |
| **Nodes positive** |  |  | <0.001 |  |  | 0.178 |
| Negative | 36 (16%) | 18 (39.1%) |  | 11 (23.9%) | 18 (39.1%) |  |
| Positive | 189 (84%) | 28 (60.9%) |  | 35 (76.1%) | 28 (60.9%) |  |
| **LODDS** |  |  | 0.019 |  |  | 0.619 |
| <1 | 88 (39.1%) | 9 (19.6%) |  | 12 (26.1%) | 9 (19.6%) |  |
| ≥1 | 137 (60.9%) | 37 (80.4%) |  | 34 (73.9%) | 37 (80.4%) |  |
